# Postprandial Changes in Thoracic Aortic Flow Detected by 4D Flow MRI May Influence Study Results

**DOI:** 10.1002/jmri.70405

**Published:** 2026-06-23

**Authors:** Maren Friederike Balks, Luisa Quest, Andreas Stroth, Alexander Reiber, Patricia Ulloa, Nicolas Kirschke, Malte Maria Sieren, Joachim Graessner, Hendrik Kooijman, Jörg Barkhausen, Alex Frydrychowicz, Thekla Helene Oechtering

**Affiliations:** ^1^ Institute of Radiology and Nuclear Medicine Universität Zu Lübeck Lübeck Germany; ^2^ Center of Brain, Behavior and Metabolism (CBBM) University of Lübeck Lübeck Germany; ^3^ Section of Pediatric Radiology, Department of Diagnostic and Interventional Radiology and Nuclear Medicine University Medical Center Hamburg‐Eppendorf Hamburg Germany; ^4^ Department of Neuroradiology Universität Zu Lübeck Lübeck Germany; ^5^ Siemens Healthineers Hamburg Germany; ^6^ Koninklijke Philips N.V Hamburg Germany; ^7^ Department of Radiology University of Wisconsin‐Madison Madison Wisconsin USA

**Keywords:** aorta, blood flow, flow quantification, phase‐contrast magnetic resonance imaging, postprandial, velocity

## Abstract

**Background:**

While there is a growing body of data using 4D flow MRI to distinguish between normal and abnormal blood flow values in the thoracic aorta, the extent of physiological variation or lack thereof is potentially undervalued. Physiological processes such as postprandial changes to hemodynamics might influence data, normal values, follow‐up studies, and data compared between study sites.

**Purpose:**

To determine the influence of food intake on flow parameters in the thoracic aorta of young healthy volunteers by 4D flow MRI.

**Study Type:**

Prospective.

**Subjects:**

20 young healthy (10 male, 10 female) volunteers were recruited for this study.

**Field Strength/Sequence:**

3T/4D flow MRI (three‐dimensional time‐resolved phase‐contrast spoiled gradient‐echo with three‐directional velocity‐encoding). Technical parameters were chosen according to consensus recommendations, including the use of a respiratory navigator, included a 10 mm respiratory navigator window, retrospective ECG gating, VENC 180–200 cm/s, voxel size 2.5 × 2.5 × 2.0–2.5 mm^3^, temporal resolution 41 ms and parallel imaging with acceleration factor of 4. Acquisition time was 10.3 ± 2.4 min.

**Assessment:**

Volunteers' thoracic aorta was imaged after fasting (food ≥ 6 h, liquid ≥ 2 h) and after a meal challenge to detect changes in aortic hemodynamics (stroke volume, flow, and velocity).

**Statistical Tests:**

Statistics included calculation of relative difference (RD), two‐tailed paired *t*‐test, and Pearson coefficient. *p* < 0.05 was considered significant.

**Results:**

4D flow MRI detected statistically significant postprandial increases in stroke volume (pre: 93 ± 18 mL; post: 99 ± 24 mL, RD = 5 ± 13%), peak flow (pre: 433 ± 95 mL/s; post: 467 ± 95 mL/s, RD = 9 ± 14%), peak average velocity (pre: 73 ± 18 cm/s; post: 78 ± 16 cm/s, RD = 8 ± 11%), and maximum velocity (pre: 118 ± 24 cm/s; post: 131 ± 23 cm/s, RD = 11 ± 11%); values given for ascending aorta. 74% of individuals presented with postprandial increase in peak flow and velocities throughout the thoracic aorta.

**Data Conclusion:**

Food intake triggers a small but significant increase in stroke volume, flow, and velocity in the aorta in young healthy individuals. Depending on the study question and potential longitudinal comparisons, one may consider introducing a fasting period prior to 4D flow MRI to avoid measurements at peak postprandial hyperemia.

**Evidence Level:**

2.

**Technical Efficacy:**

Stage 3, diagnostic thinking.

## Plain Language Summary

Food ingestion influences the entire vascular system, including the thoracic aorta. The extent of these variations is potentially undervalued and may influence study results. Four‐dimensional flow MRI was used to evaluate the effect of food intake on thoracic aortic hemodynamics in young healthy volunteers. Comparison of 4D flow derived parameters after fasting and after food intake showed small but significant increases in aortic hemodynamic parameters (stroke volume, flow, and velocity) in young healthy individuals. Considering a fasting period prior to 4D flow MRI may be helpful when planning exams to avoid peak postprandial hyperemia.

## 1 Introduction

Four‐dimensional flow MRI is a non‐invasive tool to simultaneously quantify hemodynamics and evaluate vascular anatomy in a 3D volume of interest during a single acquisition. Clinically, it is increasingly used in complex congenital cardiac pathologies to avoid misplaced or repeated 2D acquisitions and to fully capture a 3D volume uncovering hemodynamic interactions that can be missed by 2D acquisitions.

There is debate about the impact of food intake on aortic hemodynamics. While 4D flow MRI can be used to quantify changes in hepatic hemodynamics after a meal [1], the impact on aortic hemodynamics remains less well understood. Food intake increases blood flow to digestive organs to facilitate nutrient absorption [2]. Meal challenges combined with MRI can be used to detect disorders, such as a blunted mesenteric hyperemia due to superior mesenteric artery stenosis or portal hypertension [1, 3, 4, 5, 6, 7, 8]. However, food ingestion not only affects the gastrointestinal tract but also elevates cardiac output, heart rate, and vascular resistance [2, 9, 10]. Food intake thus influences the entire vascular system, including the thoracic aorta. However, the magnitude of these changes is unclear.

There is a lack of knowledge regarding whether a fasting period should be implemented prior to 4D flow MRI of the thoracic aorta to ensure comparability of data between studies or in follow‐up examinations. Therefore, the purpose of this work is to evaluate the capability of 4D flow MRI to detect and quantify the effects of food intake on thoracic aortic hemodynamics in young healthy volunteers.

## 2 Methods

### 2.1 Subjects

The local ethics committee has approved this HIPAA‐compliant study (AZ17‐252). All participants provided written informed consent prior to enrollment, following a comprehensive explanation of the study.

For this prospective study, volunteers between 18 and 40 years of age were recruited (*n* = 20, 50% male, 50% female). Exclusion criteria included known cardiac illness, contraindication for MRI examinations, and pregnancy. All volunteers were scanned after fasting (no food for at least 6 h, no liquids for at least 2 h), and again 30 min after a meal. For the meal challenge, subjects were asked to eat a meal with a nutritional score of 600–700 kcal [6, 11] within 10 min. Carbohydrate or fat content of the meal was not controlled in this pilot study. Volunteers were given time to acclimate to the scanner environment before the MRI exams to minimize stress‐induced hemodynamic changes. Analysis of study collective data included volunteers' age, height, weight, and derived BMI.

### 2.2 MRI Examination

Participants were randomly assigned to one of the MRI 3T systems for both of their scans, either Ingenia (Koninklijke Philips N.V., Netherlands) with a 20‐channel body surface coil or MAGNETOM Skyra (Siemens Healthineers, Germany) with an 18‐channel body surface coil. Coil sensitivity calibration for parallel imaging was performed following vendor recommendations. 4D flow images were a**c**quired following consensus statement recommendations [12, 13] that were carefully adjusted and tested [8] to closely match between systems. Typical technical parameters are summarized in Table 1. We used ECG‐based gating with VCG (vectorcardiogram) triggering for retrospective cardiac gating. Volunteers were instructed to breathe freely and a respiratory navigator with a 10 mm acceptance window was placed on the liver/lung interface for motion compensation.

**TABLE 1:** Technical parameters of 4D flow sequence at 3T (Ingenia, Koninklijke Philips N.V., Netherlands and MAGNETOM Skyra, Siemens Healthineers, Germany).

	Philips	Siemens
Repetition time (ms)	3.6	3.9
Echo time (ms)	1.58	2.28
Flip angle (°)	8	8
Velocity encoding (VENC) (cm/s)	180–200	180–200
Width of respiratory navigator (mm)	10	10
Cardiac gating	Retrospective	Retrospective
Cardiac time frames (depending on heart rate, adjusted so that the reconstructed temporal resolution matched closely to true temporal resolution)	17–35, resulting in a mean temporal resolution of 40 ms (range 36–48 ms)	17–35, resulting in a mean temporal resolution of 42 ms (range 29–58 ms)
Number of slices	26–34	26–34
Voxel size (mm^3^)		
Acquired	2.5 × 2.5 × 2.5	2.5 × 2.5 × 2
Reconstructed to	2 × 2 × 2	2 × 2 × 2
Parallel imaging (acceleration factor)	SENSE (4)	GRAPPA (4)

### 2.3 Data Processing

Reconstruction of raw data was performed on the MRI system to obtain phase and magnitude images (online reconstruction). The duration of the reconstructed cardiac phases per R–R interval was adjusted to closely match the true temporal resolution of the MRI sequence. This was achieved by adjusting the number of cardiac time frames according to the individual's heart rate (Philips: “actual phase” should be close to 100%; Siemens: average RR − interval divided by effective TR). Inline post‐processing included correction for concomitant gradient fields (Maxwell terms). Philips inline postprocessing also included automatic correction for background phase offsets due to eddy currents. For all Siemens data, correction of background phase offsets due to eddy currents was performed using GTFlow v3.2.15 (Gyrotools LLC, Zurich, Switzerland).

### 2.4 Evaluation of Quantitative Parameters

**FIGURE 1:**
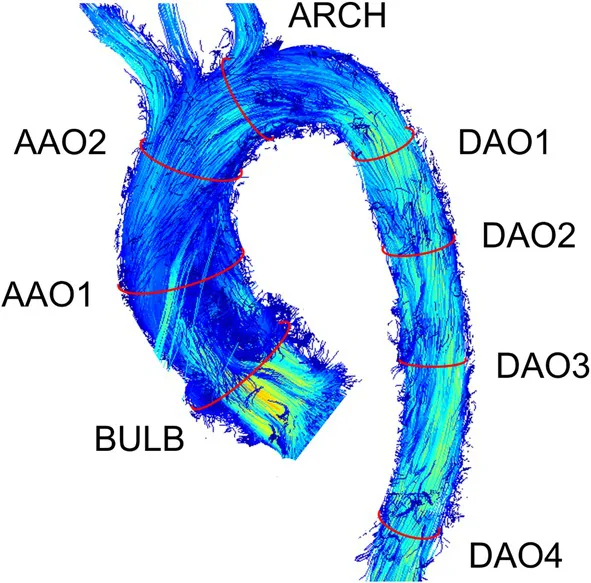
Contours were placed manually at the following landmarks: (1) at the aortic bulb (BULB), (2) in the proximal ascending aorta on the level of the pulmonary trunk (AAO1), (3) in the distal ascending aorta proximal to the brachiocephalic trunk (AAO2), (4) in the aortic arch between the right subclavian artery and the left common carotid artery (ARCH), (5) in the descending aorta at the level of AAO2 (DAO1), (6) in the descending aorta on the level of the pulmonary trunk (DAO2), (7) in the descending aorta on the level of the aortic bulb (DAO3), and (8) in the descending aorta just above the diaphragm.

All data were transferred in DICOM format to an offline computer. Evaluation of quantitative parameters was performed using GTFlow v3.2.15 (Gyrotools, Switzerland) on the following eight contours perpendicular to the vessel wall and main flow direction (Figure 1): aortic bulb (BULB), ascending aorta (AAO1, AAO2), aortic arch (ARCH), and descending aorta (DAO1–DAO4). Contour adjustments for each time frame were carried out manually to account for motion of the aortic wall during the cardiac cycle. Segmentation was performed by co‐authors AR and LQ as part of their doctoral thesis, both had 1 year of experience with 4D flow MRI. This study focused on parameters routinely used in clinical settings to quantify shunt (net stroke volume, SV, mL per cardiac cycle), valve insufficiency quantification (forward and backward stroke volumes, mL per cardiac cycle), and for stenosis (maximum velocity, *V*_max_, cm/s). Net stroke volume represents the effective volume of blood passing through a vessel per cardiac cycle, accounting for both forward and reverse (=backward) flow. It is calculated as: net SV = forward SV − backward SV. Forward stroke volume is defined as the volume of blood moving in the physiological direction. Backward stroke volume is the volume of blood regurgitating in the opposite direction due to valve insufficiency. Maximum velocity is defined in GTFlow as the peak velocity of a single voxel in the contour. Being prone to error due to noise [8], we added the peak of the average velocity per contour (peak average velocity, *V*_el_, cm/s) to the analysis. Moreover, we included peak flow (flow, mL/s) and average contour area (area, mm^2^), as they are both used to calculate the stroke volume and could be affected differently by a meal challenge. Volunteers’ heart rate was recorded from the 4D flow data of the fasting and postprandial exam.

### 2.5 Statistics

To account for potential variability in the placement of AAO and DAO cut planes, in order to decrease influence from noise, the average of multiple cut planes within vascular segments was used in accordance with the recommendations of the Quantitative Imaging Biomarker Alliance (QIBA) [14].

Hemodynamic parameters and heart rate during the exam after fasting were compared to the results after food intake. Kolmogorov–Smirnov test was used to test for normal distribution. In case of normal distribution, the hypothesis was tested by comparison of average values using two‐tailed paired tailed *t*‐test; otherwise, a Wilcoxon‐test was performed. Relative difference of the hemodynamic parameters was determined as [(postprandial result − fasting result)/fasting result]. Results are given in as mean (standard deviation) %.

Forty‐five percent of data chosen randomly were analyzed twice by two different readers to evaluate interreader‐variability. Both readers placed contours independently. Results are given as absolute difference (mean ± standard deviation), and relative difference [(2nd reader result − 1st reader result) divided by mean; mean ± standard deviation as percentage %]. The Pearson coefficient was calculated to test the linear correlation between readers.

*p* < 0.05 was considered significant. Microsoft Excel (Microsoft Excel, Microsoft 365 MSO 32‐bit) and SPSS (IBM SPSS Statistics Subscription, version 2021) were used for statistical analysis.

## 3 Results

### 3.1 Study Collective

Twenty healthy subjects were examined before and after a meal challenge using 4D flow MRI. One data set had to be excluded due to a technical error when storing the data which prevented further analysis. Accordingly, 4D flow MRI of 19 volunteers (10 male, 9 female) after fasting and after a meal challenge were analyzed, resulting in a total of 38 datasets. All volunteers were within a focused age group (mean age 25.4 ± 3.5 years, range 21–32 years). Their BMI was 23.0 ± 2.5 and did not differ significantly (*p* = 0.79) between male and female participants (Table 2). Individual heart rate during the 4D flow exams increased in 68% of volunteers after the meal challenge. This did not reach statistical significance (pre: 64 ± 13 bpm, post: 66 ± 13 bpm; *p* = 0.3). 4D flow MRI acquisition time was 10.3 ± 2.4 min (mean ± SD, range 7–16 min). Time between meal challenge and the start of the postprandial 4D flow sequence was within 30 min, which is within the reported time of maximum postprandial response between 30 and 60 min [15, 16, 17].

**TABLE 2:** Study collective data of healthy volunteers.

	Total (*N* = 19)		Men (*N* = 10)		Women (*N* = 9)		*p*
	Mean	SD	Mean	SD	Mean	SD	
Age (years)	25.3	3.6	26.2	4.3	24.6	3.0	0.44
Weight (kg)	73.0	9.9	77.6	9.5	67.4	7.4	***0.02***
Height (cm)	178.2	9.0	183.0	7.3	172.2	7.4	***< 0.005***
Body mass index (BMI)	23.0	2.5	23.1	2.2	22.8	3.0	0.79
Heart rate (bpm)							
After fasting	64.7	13.1	66.3	14.2	62.5	11.0	0.25
Postprandial	67.1	13.4	65.0	14.6	70.0	11.0	0.26

*Note:* All data are given as mean and standard deviation (SD). Significant differences are marked in bold italics.

### 3.2 Quantitative Hemodynamic Parameters

**FIGURE 2:**
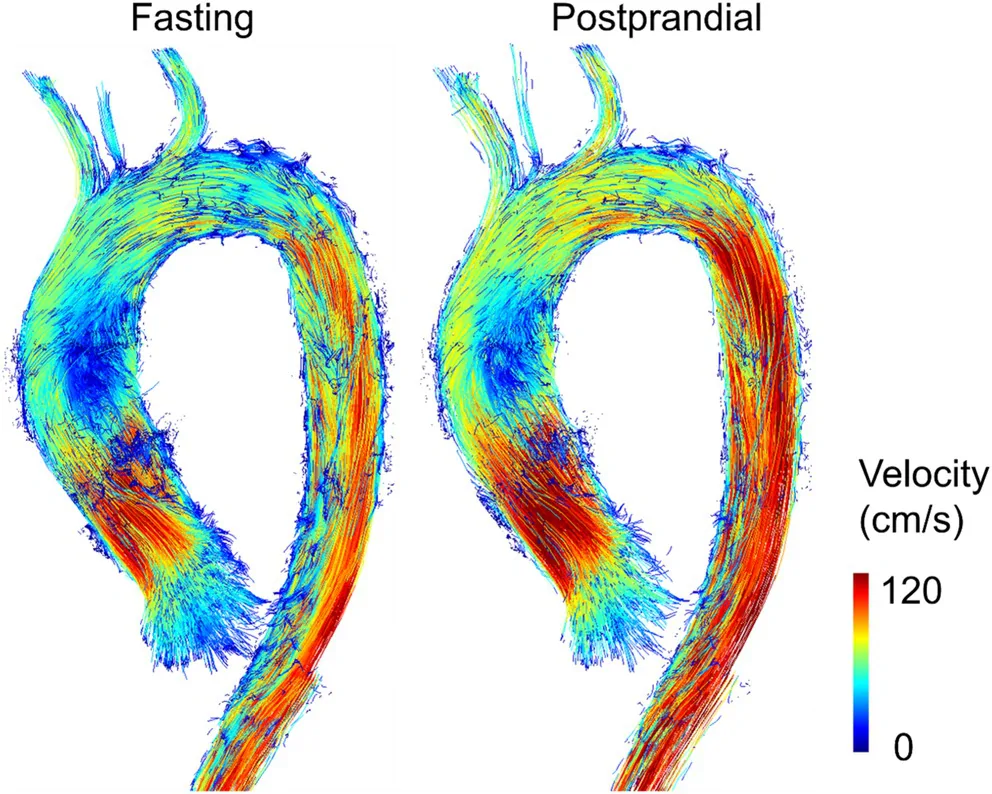
Particle trace visualization of aorta hemodynamics of a 30‐year‐old male shows an increase in flow velocity in the entire aorta after the meal challenge (right) compared to the fasting exam (left).

**FIGURE 3:**
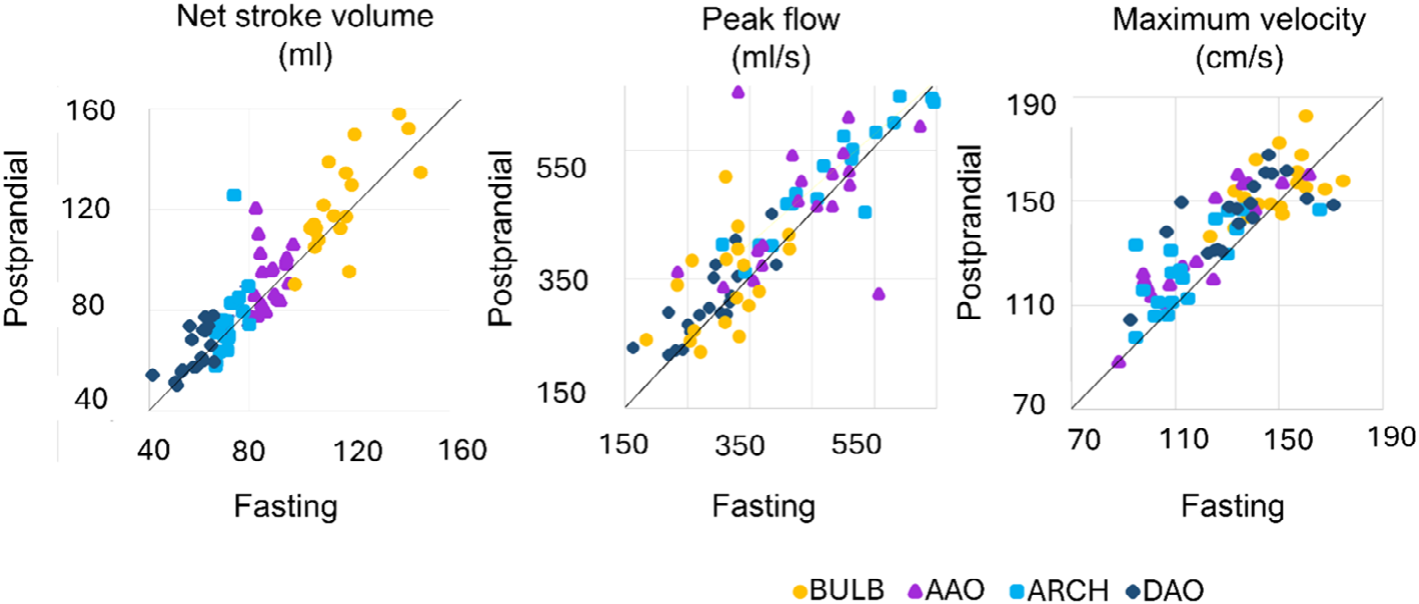
Net stroke volume, peak flow, and maximum velocity of all 19 individuals before and after the meal challenge. Food intake increased all parameters for most individuals at the aortic bulb (BULB), the ascending aorta (AAO), the aortic arch (ARCH), and the descending aorta (DAO). Only at the aortic bulb and in the aortic arch, net stroke volume did not increase significantly postprandial.

Four‐dimensional flow MRI detected a increase in thoracic aortic peak flow, average velocity, maximum velocity, as well as net stroke volume after food intake compared to the exam after fasting: Peak flow (pre: 433 ± 95 mL/s; post: 467 ± 95 mL/s, RD = 9 ± 14%), peak average velocity (pre: 73 ± 18 cm/s; post: 78 ± 16 cm/s, RD = 8 ± 11%), and maximum velocity (pre: 118 ± 24 cm/s; post: 131 ± 23 cm/s, RD = 11 ± 11%) were significantly higher after food intake (numbers given as examples for ascending aorta, detailed results are listed in Table 4). The same was true for net stroke volume in the ascending (pre: 93 ± 18 mL; post: 99 ± 24 mL, RD = 5 ± 13%) and descending aorta (pre: 65 ± 13 mL; post: 72 ± 18 mL, RD = 11 ± 18%). In general, 74% of individuals presented with postprandial increase in all of the following parameters in the entire thoracic aorta: peak flow, peak average velocity, and maximum velocity (Table 3, also Figures 2 and 3). Mean relative increases ranged between 2% and 11% for all hemodynamic parameters and contours.

**TABLE 3:** Comprehensive results of pre‐ and postprandial scans.

	Fasting			Postprandial			Subjects (%) with postprandial increase	Relative difference (%)		
	Mean	SD	Range	Mean	SD	Range		Mean	SD	*p*
Net stroke volume (mL)										
BULB	105.8	23.1	69.2–148.4	107.3	24.0	76.2–158.0	58	2	9	0.55
AAO	93.3	17.8	56.1–122.0	98.5	24.4	69.4–139.0	58	5	13	***0.02***
ARCH	70.1	13.5	41.3–104.3	76.7	21.2	54.3–125.7	72	9	20	0.06
DAO	64.9	12.9	37.6–84.3	71.9	18.4	50.1–123.4	74	11	18	***< 0.001***
Peak flow (mL/s)										
BULB	484.9	99.5	305.8–645.0	519.0	93.8	361.1–694.3	84	8	10	***< 0.001***
AAO	432.9	95.1	234.0–556.9	466.7	95.0	327.2–640.2	74	9	14	***< 0.001***
ARCH	307.2	59.6	183.4–413.2	334.6	75.2	237.5–509.7	74	9	12	***0.01***
DAO	283.9	57.1	163.0–392.8	311.7	61.7	232.9–451.7	79	11	15	***< 0.001***
Peak average velocity (cm/s)										
BULB	70.6	7.8	50.0–82.8	74.6	5.6	61.6–84.9	79	6	9	***0.01***
AAO	73.1	18.2	38.4–108.4	78.2	15.6	54.3–104.1	79	8	11	***< 0.001***
ARCH	74.4	13.2	42.4–104.9	79.8	10.4	58.1–95.0	74	9	12	***0.01***
DAO	87.0	13.8	70.6–111.5	92.3	10.9	70.9–106.3	74	7	12	***< 0.001***
Maximum velocity (cm/s)										
BULB	148.3	13.3	123.5–174.6	154.5	11.6	136.4–182.8	74	5	8	***0.04***
AAO	118.0	23.6	67.5–161.5	130.7	23.0	88.6–160.2	84	11	11	***< 0.001***
ARCH	113.3	20.9	66.5–165.9	122.5	17.9	84.0–146.9	79	9	12	***< 0.001***
DAO	134.1	21.8	85.3–170.7	145.3	18.8	109.6–167.7	79	9	14	***< 0.001***
Average contour area (mm^2^)										
BULB	781.9	149.5	528.2–1122.8	771.6	145.8	563.0–1027.8	26	1	7	0.45
AAO	610.6	155.4	423.2–923.0	612.6	163.1	436.4–933.9	42	0	5	0.96
ARCH	423.5	88.0	291.5–682.7	421.5	88.8	312.6–679.0	42	0	7	0.81
DAO	330.9	92.04	259.2–618.7	336.0	90.6	245.0–616.3	53	2	8	0.21

*Note:* Results are given as mean and standard deviation (SD) as well as range. How many subjects presented with a postprandial increase of hemodynamic parameters are given as a percentage (%). Significant differences are marked in bold italics. Ascending aorta (AAO) and descending aorta (DAO) include the averaged values of the contours.

Interreader‐results showed excellent correlation. Mean relative bias between two readers was at or below 3% for all parameters (Table 4).

**TABLE 4:** Comparison of interreader variability of circadian evaluation for the total aorta combined.

	Absolute difference		Relative difference (%)		Correlation
	Mean	SD	Mean	SD	*r*
Stroke volume (mL)	0.9	4.6	2	7	0.98
Peak flow (mL/s)	4.5	16.2	2	6	0.99
Peak average velocity (cm/s)	−2.4	5.8	3	8	0.92
Maximum velocity (cm/s)	0.4	10.7	0	8	0.89
Average contour area (mm^2^)	2.8	64.0	1	8	0.93

*Note:* Results of absolute difference are given as mean ± standard deviation (SD), relative difference as mean ± SD in %.

## 4 Discussion

Postprandial variations in thoracic aortic flow parameters were detected using 4D flow MRI, with relative increases for stroke volume, peak flow, peak average velocity, and maximum velocity after a meal. These findings demonstrate the sensitivity of 4D flow MRI to physiological changes induced by food intake in young healthy individuals.

Postprandial changes in thoracic aorta hemodynamics are consistent with known cardiac adaptations, including increased cardiac output and splanchnic blood flow after a meal [18, 19]. Prior phase MRI studies have shown similar effects in hepatic and mesenteric circulation [1, 6, 20], but data on thoracic aortic response have been limited.

The increase in cardiac output was described to be mainly due to increased stroke volume and heart rate [21]. Although over two thirds of study participants experienced an increase in heart rate, this increase did not reach statistical significance in our cohort of young healthy volunteers. However, the net stroke volume in the thoracic aorta increased significantly as expected. As we only examined healthy young subjects, we hypothesize that the increase in stroke volume was enough with the modest increase in heart rate to accommodate the increased cardiac output required for postprandial status [10, 22]. Increased stroke volumes in the aorta after a meal may impact absolute shunt volume quantification. Furthermore, postprandial increase in peak velocity could distort grading of stenosis in clinical routine. This effect could be mitigated by giving shunt fractions in percent in follow‐up exams. Setting reference values for physiological stress such as food ingestion is useful to contextualize changes in hemodynamics in the thoracic aorta. The variability of hemodynamics after food intake in our subjects was significant. Depending on the parameter, the changes were similar to or exceeded previously reported test–retest variability: Van Ooij et al. [23] reported a test–retest variability of 8% for velocity in the aorta of healthy volunteers, which is similar as compared to the increase we detected after a meal. Markl et al. [24] described differences < 5% for test–retest for flow and peak systolic velocity in the aorta of healthy volunteers, which is lower than the detected postprandial increase found in our study.

Repeatability of 4D flow MRI may be reduced by technical as well as physiological variability. Technical variability can be introduced, for example, by different sequences, sequence settings, as well as differences in postprocessing workflow and software. In contrast, physiological variability of hemodynamics is introduced by the patient's normal physiological response to different events such as food intake. To optimize reproducibility between exams, the extent of physiological variability can be mitigated once relevant influencing factors are identified. This study identified food intake as a significant modifier of hemodynamics. Standardizing examination conditions can help improve the reproducibility and thus the value of 4D flow MRI follow‐up studies. Diet recommendations prior to the MRI examination are feasible in clinical practice and are already in place in many institutions for contrast‐enhanced exams. A frequently implemented rule, for example, is fasting 2 h prior to the exam to minimize complications if the patient develops nausea from the contrast injection. This would accommodate results by Waaler et al. [25] who described a return of cardiac output to fasting levels within 2 h after food intake. However, the cardiac response to a meal also depends on age as well as on the composition and size of a meal [21, 25], and the value of a 2‐h fasting recommendation should be validated in future studies. Past work has shown that in young subjects, blood pressure remains the same after a meal high in carbohydrate as well as after a meal high in fat share [26]. In patients with pre‐existing cardiac diseases, however, even a small meal can aggravate their clinical condition. This effect has been described as early as 1772, when Heberden [27] reported the exacerbation of angina pectoris after food intake. Hence, it would be interesting to investigate the hemodynamic effect of a food challenge in patients with preexisting cardiac disease in comparison to the here presented young healthy cohort.

### 4.1 Limitations

This pilot was limited to a small cohort of young healthy volunteers, a cohort in which the most hemodynamic change is expected, to confirm if hemodynamic difference after a meal in the aorta where measurable at all. Hence this work forms the basis for future studies including older populations and patients with cardiac disease to evaluate whether impaired postprandial adaptation can serve as a diagnostic marker. Moreover, we did not assess the impact of different meal compositions, fluid intake. Investigating the effects of meal composition and timing may further refine preparation recommendations. Further, we did not include a repeat exam to confirm normalization of hemodynamics after the meal challenge nor did we do two fasting exams to exclude time related changes. A discussion of general limitations of the method of 4D flow MRI can be found in Bissel et al. [13].

## 5 Conclusion

We demonstrated that changes in aortic hemodynamics after food intake can be detected by 4D flow MRI in a small cohort of healthy volunteers. Net stroke volume, peak flow, peak average velocity, and maximum velocity in healthy subjects were on average 2%–11% higher after a meal than after fasting. Our results align with previous 4D flow MRI studies that demonstrated postprandial mesenteric hyperemia. However, the observed changes in thoracic aortic flow parameters extend this knowledge and highlight the systemic nature of postprandial hemodynamic shifts. Acknowledging the small but measurable impact of a meal on the thoracic hemodynamics in healthy young individuals is an important first step; future studies should focus on identifying differences between age groups as well as between patients and healthy individuals. Detecting impaired adaptation to food intake could help with cardiac risk stratification. Postprandial changes were small scale but measurable. There are many potential influencing factors on hemodynamics that can add up. Postprandial variability is a factor that can be controlled relatively easily. If very precise measurements for follow‐up exams are required, a fasting period of at least 2 h prior to MRI can be considered. This is clinical standard at many centers for routine MRI exams already.

## Author Contributions

T.H.O., A.F., and J.B. conceived of the study. J.G., P.U., and H.K. participated in the study design and setup of CMR protocols. L.Q., A.S., A.R., N.K., M.M.S., and T.H.O. conducted CMR studies, data analysis, and data interpretation. M.F.B., L.Q., and T.H.O. performed statistical analyses. J.G. and H.K. were not involved in data analysis and interpretation to exclude competing interests. M.F.B., L.Q., and T.H.O. drafted the manuscript. All authors read and approved of the final manuscript.

## Funding

This work was supported by Universität zu Lübeck (AZ17‐252 and J31‐2018).

## Ethics Statement

Positive approval by the ethics committee of Universität zu Lübeck has been received (AZ17‐252).

## Consent

A written study consent was signed by all volunteers after being informed about the study.

## Conflicts of Interest

There were research collaborations between the Institute of Radiology and Nuclear Medicine, Universität zu Lübeck, Germany with the scanner and software vendors mentioned in this paper: Gyrotools, Switzerland; Koninklijke Philips N.V, Netherlands; Siemens Healthineers, Germany (alphabetic order). H. Kooijman worked for Philips. J. Graessner worked for Siemens. The other authors declare that they have no competing interests.
